# Activating the Anaphase Promoting Complex to Enhance Genomic Stability and Prolong Lifespan

**DOI:** 10.3390/ijms19071888

**Published:** 2018-06-27

**Authors:** Troy A. A. Harkness

**Affiliations:** Department of Anatomy and Cell Biology, University of Saskatchewan, Saskatoon, SK S7N 5E5, Canada; troy.harkness@usask.ca; Tel.: +1-(306)-966-1995

**Keywords:** Anaphase Promoting Complex, lifespan, cancer, yeast, human cell culture

## Abstract

In aging cells, genomic instability is now recognized as a hallmark event. Throughout life, cells encounter multiple endogenous and exogenous DNA damaging events that are mostly repaired, but inevitably DNA mutations, chromosome rearrangements, and epigenetic deregulation begins to mount. Now that people are living longer, more and more late life time is spent suffering from age-related disease, in which genomic instability plays a critical role. However, several major questions remain heavily debated, such as the following: When does aging start? How long can we live? In order to minimize the impact of genomic instability on longevity, it is important to understand when aging starts, and to ensure repair mechanisms remain optimal from the very start to the very end. In this review, the interplay between the stress and nutrient response networks, and the regulation of homeostasis and genomic stability, is discussed. Mechanisms that link these two networks are predicted to be key lifespan determinants. The Anaphase Promoting Complex (APC), a large evolutionarily conserved ubiquitin ligase, can potentially serve this need. Recent work demonstrates that the APC maintains genomic stability, mounts a stress response, and increases longevity in yeast. Furthermore, inhibition of APC activity by glucose and nutrient response factors indicates a tight link between the APC and the stress/nutrient response networks.

## 1. Introduction

When does the aging process begin? How long can we live? Why do we age? These questions are highly debated with no distinct, definitive answers. Does aging begin when our skin starts to wrinkle, or when our hair commences to turn grey? Or perhaps aging begins after the completion of growth [1]. Aging has also been defined as a shift in an organism’s aging reality. The aging reality has been described as a mutually enslaved system of DNA and its environment in which signaling failures within this DNA environment occur over time [2]. Much of the debate reflects the fact that we have not perceived children as aging; however, acquired somatic mutations are recognized in infants and children, leading to the development of childhood cancers [3,4,5]. More compelling support for childhood aging comes from premature aging syndromes, such as progeria, in which children age in a very similar manner to normal aging individuals but at an 8-fold accelerated rate [6]. Even Hayflick considered when aging begins, weighing the possibilities that aging starts before or at conception or, alternatively, when maximum strength and stamina is achieved [7]. Hayflick eventually concluded that aging is a stochastic program that begins after reproductive maturity in animals, resulting in the loss of molecular fidelity. This loss of fidelity ultimately surpasses repair capacity, leaving individuals prone to age-related diseases [8,9]. The idea that aging is a random stochastic program is supported by many researchers in the field [10,11]. The stochastic idea of aging gained traction when the free radical theory of aging was proposed. This theory states that aging occurs due to the natural wear and tear of cellular machinery and biological substances due to exposure to free radicals generated within the cell [12]. Biological systems are constantly fighting a battle with its environment, both internally and externally, to ward off damage. The simple generation of mitochondrial-dependent energy and DNA replication expose cells to damage that must be repaired. Evidence for a stochastic program of aging also comes from inorganic compounds that age over time; for example, rusting of metal and peeling of paint (discussed in [13]), implying that something beyond genetics controls aging. From this work comes the idea that entropy drives aging, while genes drive longevity.

This review will summarize the current ideas describing our thoughts on the aging process. Molecular mechanisms will then be described that facilitate cellular aging. The notion that genomic instability is the driving force leading to aging and age-related disease will be discussed. Finally, the novel concept that activation of a cell cycle regulator, the Anaphase Promoting Complex, which is required for maintenance of cell health, inhibition of cancer progression, and enhanced longevity, will be explored.

## 2. Genetic Control of Longevity

Longevity depends on how long our cells remain functional, which is countered by the many insults faced by cells. Pathways that maintain cellular homeostasis are genetically controlled; thus, it clearly follows that a genetic program would be in place to control longevity. A recent review links proteostasis (a housekeeping phenomenon that controls the integrity of protein structure and function) with lifespan determination, and suggests the failure of the proteostatic network occurs early in life and marks the beginning of aging [14]. A global network encompassing maintenance of genomic stability, as well as enhancing proteostasis, would involve, for example, genes and proteins that repair DNA, scavenge free radicals, and the proteins that run these programs. Thus, the genetic and stochastic models appear to oppose one another. On the one hand, the stochastic model dictates that over time, our cells randomly accumulate damage, such as the accumulation of DNA mutations and genomic instability, and eventually succumb to the damage. On the other hand, the genetic program is in place to provide cells with options to survive the intrinsic and extrinsic environmental assaults that chronically bombard the cell and the biological system as a whole. Evolutionary biologists have argued that selection of lifespan-extending genes is unlikely, since the effects of these genes would only be seen long after reproduction has ceased, with the force of natural selection that declines with age leaving no valid reason to remain alive [2,15]. However, when these genes are viewed as controlling cell repair in the face of a damaging environment in order to survive, then it becomes clear that enhanced longevity may only be an indirect benefit accompanying the ability to survive unfavorable life events. Thus, evolution may not be selecting for longevity genes at all, but simply looking for genes that increase survival under trying times, with increased longevity simply a lucky side effect. This idea forms the foundation of the theory describing the response to nutrients versus stress as a driving force defining one’s lifespan, as suggested earlier [16]. The concept of Hormesis, in which a potentially life-threatening stress, when given in a low dose, increases health or extends lifespan, is an example of a stress response providing a favorable and beneficial reaction [17,18].

One genetic theory of aging that is appealing to evolutionary biologists is the idea of pleiotropic antagonism. Antagonistic pleiotropy suggests that genes that are beneficial in the early years become harmful in later life [19,20,21]. A similar idea is described in the Disposable Soma theory [15]. It was proposed that because of high environmental mortality, resources are primarily spent on growth and reproduction, rather than on the soma, which would leave the soma exposed to environmental and intrinsic stresses. Nonetheless, the idea has been used to describe a Darwinian-evolutionary concept of aging in which the deleterious effects of previously beneficial genes in later life leads to the development of age-related disease [22]. Examples of antagonistic pleiotropy and how it could contribute to a Darwinian-evolutionary concept include the calcification of bones, which enables strength in early life, but eventually leads to deleterious calcification of arteries in late life. The erosion of telomeres, often considered a clear mechanism of aging, is also considered as support of a Darwinian-evolutionary model. While expression of telomerase extends telomere length in stem cell populations, it also contributes to tumor formation [23]. Thus, in early life, longer telomeres maintain the health and vitality of the cell. However, in later life, the inactivation of telomerase is proposed to ward off cancer, but at the cost of continued telomere erosion, and likely cellular senescence [24]. Darwinian selection of stress sensing and Darwinian selection of antagonistic pleiotropy genes are both used as examples of alternative mechanisms of lifespan determination, as both provide benefits in early life. However, the selection of stress sensing genes, but not antagonistic pleiotropy genes, provides an indirect longevity benefit in later life.

## 3. Genetic Control of Aging

The genetic model of lifespan determination is not at odds with the stochastic model, and involves a genetic program that determines the length of time that our cells and bodies can function. In the end, the more capable the cell is at damage repair, occurring through stochastic intrinsic and extrinsic events, the better the odds of surviving harsh environments and living to pass on genes to descendants. Longevity of the organism starts with the health of the cells. If cell health cannot be maintained, then health of the tissues and the animal itself will suffer. Cell type, in particular, is determined by programmed genetic and epigenetic networks. In the human body, for example, each cell harbors the same sequence of DNA, yet different cells carry out very different functions. Alterations to any of these networks can signal the end to that cell. Maintenance of the cellular equilibrium supporting tissue renewal is critical to the longevity of the organism. Over time, equilibrium and cell renewal begins to fail, leading to reduced replacement of cells lost due to attrition or senescence. Thus, the failing of the genetic system, contributing to the loss of cell equilibrium and renewal through accumulating mutations, is considered a hallmark of aging. Hallmarks of aging consist of the following attributes: genomic instability, telomere attrition, epigenetic alterations, and loss of proteostasis, leading to deregulated nutrient sensing, altered mitochondrial function, and cellular senescence [25,26].

Specific genes, many of which were first described and characterized in the simple lower eukaryotic yeast system (i.e., *SIR2* (yeast *SIRT* gene), *SNF1* (yeast *AMPK*), *FKH1*/*2* (yeast *FOXO*), *SCH9* (yeast *AKT*/*S6K*), *TOR1*, and *RAS2*, have been shown to be evolutionarily conserved genes that respond to stress or nutrients, influencing longevity [27,28,29,30,31,32]. Thus, genes (such as *SIR2*) clearly play a significant role in promoting lifespan from single cells to humans, but do genes also drive the loss of homeostasis and the aging process? While the stress response genes drive cell health and longevity in the presence of low-level stress, an example of Hormesis [17,18], the nutrient response genes do the opposite, and act as pro-aging genes. Thus, genes provide the impetus for both longevity and aging. Unlike the stochastic model, which relies on random factors to drive the aging process, the genetic program promoting aging relies on the activation of a web of nutrient response genes that inhibit the stress response network in the presence of usable resources [33]. Lifespan extension through caloric restriction is a classic example of Hormesis, and can be mimicked in yeast by mutating genes, such as *SCH9*, *TOR1,* or *RAS2*, which encode nutrient response proteins [29]. Ultimately, the longevity of an organism depends on the effectiveness of the counterbalanced stress and nutrient sensing pathways.

## 4. A Ceiling on a Maximum Lifespan?

Support for a predetermined program delineating our lifespan has been around for decades and is derived from the knowledge that human mean lifespan has increased dramatically over the past century, but the maximum lifespan has not [34]. The longest verified living human, Jeanne Calment, died at age 122 in 1997 [35], which is consistent with the idea that the maximum attainable human lifespan is not increasing and is likely capped at 125 years [36,37]. Indeed, few humans have ever lived past 115 years [38]. A maximum of 125 years is nonetheless controversial, as some feel there is no limit to our lifespan [8,39,40,41,42,43]. Further evidence supporting the idea that a predetermined genetic program dictates our maximum lifespan is provided by observations that maximum achievable lifespans are observed across evolutionary boundaries, as each specific organism seems to have a built in maximum possible lifespan [44]. The ever-increasing mean lifespan observed over the past century has also been used as evidence that human maximum lifespan will also continue unimpeded. Of course, this rise in expected lifespan is largely attributable to new developments in medical care, improved diet, less exposure to toxins, and regular exercise, which may only increase healthspan, and not lifespan. Regardless of how people feel about the dramatic rise in global expected lifespans, time is needed to fully realize the effect of improved human well-being, and thought should be put into policy development to deal with the likelihood that people will be living longer, healthier lives. Thus, if maximum lifespans have reached a ceiling, with mean life expectancy continuing to rise, lifespan curves may soon be considered lifespan cliffs, with increased human productivity a likely benefit.

## 5. So When Does Aging Begin?

It now seems quite clear that cellular aging is largely dependent on the degree to which genomic instability has affected DNA-dependent processes. Many studies, from yeast to humans, have repeatedly shown that during aging, senescent cells that exit the cell cycle or cease to function harbor large accumulations of DNA mutation, rearrangements, and epigenetic alterations. There are numerous sources of DNA damage, both endogenous and exogenous, that the cell must deal with. It is thought that a somatic cell may receive as many as 100,000 lesions daily [45,46]. It is not a coincidence that most age-dependent diseases, such as cancer, type II diabetes, and cardiopulmonary and neurodegenerative diseases are associated with increasingly elevated levels of genomic instability that occur over time [47,48,49,50,51]. When a cell is born, it is presumably at its functional apex, performing at its highest level. In yeast, the mother cell sequesters damage so that the daughter does not receive it, having a much better chance to begin life in a pristine state [52,53]. However, eventually the damage is too much for the yeast mother cell to fully sequester, with the daughter born with accumulating damage. If similar mechanisms that occur in yeast are occurring in higher eukaryotic systems, then it is easier to understand how a newly born cell would be at its best to repair damage and maintain proteostasis. With this in mind, the answer for when aging begins might be when the cells that form the zygote are first born; thus, aging of an individual may begin much earlier than conception, such as at the very moment when the mother develops oocytes in utero [54].

## 6. Connecting Stress Sensing with Nutrient Sensing

Genomic instability appears to be the gateway to aging and age-related disease. Genomic stability is threatened as soon as a cell is born due to the intrinsic damage caused by energy generation and the errors inflicted by DNA replication. The damage repair processes are presumably functioning at their best in these new cells, so genomic instability likely does not become an obstacle until much later in life. As discussed above, multiple antagonistic molecular networks are vying for available resources to respond to either stress and/or nutrients. It should be clear that the opposition of these pathways should not be all or none, as aspects of nutrient availability may be present even in an unfavorable environment. Thus, the question becomes how are nutrient and stress sensing networks regulated? What mediates the end of stress signaling when the stress is gone, or the stalling of the nutrient sensing pathways when the food source is used up?

## 7. The Anaphase Promoting Complex, Using Chromatin Assembly during Mitosis to Maintain Genome Stability

**Figure 1:**
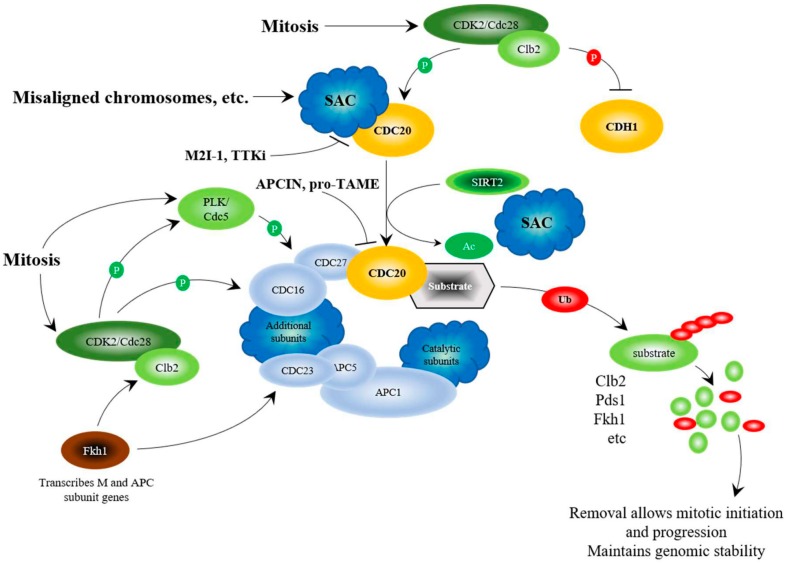
Regulation of the APC at the G2/M transition. The schematic considers results from mammalian and yeast studies. Yeast proteins are written as Cdc20, whereas mammalian proteins are written as CDC20. Genomic stability and segregation of replicated and repaired chromosomes is established via the Spindle Assembly Checkpoint (SAC) that sequesters Cdc20/CDC20 away from the APC, thus inhibiting APC function as cells enter mitosis [77,78]. When the SAC is satisfied, the cyclin Clb2 (Cyclin B), synthesized during G2 by Fkh1 [79], interacts with cyclin-dependent kinase Cdc28 (CDK2) to phosphorylate a series of proteins needed for mitotic progression: Cdc5 (PLK), Cdc16, Cdc23, and Cdc27 [80,81]. Once PLK is active, it further activates the APC by phosphorylating Apc9 (or APC1 in mammalian cells), Cdc16 and Cdc27 [80,81]. Cdc28-Clb2 also phosphorylates the co-activators Cdc20 for activation [80], and Cdh1 for inhibition [82]. A further activating stimulus is provided by SIRT2, which deacetylates CDC20 [83]. APC^Cdc20^ then targets proteins for degradation, such as Pds1 (PTTG1/Securin), to allow chromosome segregation, and Clb2 and Fkh1 to complete a negative feedback loop that prepares the cell for mitotic exit and G1 maintenance [65,84,85]. Degradation of Clb2 stops inhibition of Cdh1, allowing replacement of the APC^Cdc20^ complex with APC^Cdh1^. APC chemical inhibitors, APCIN and pro-TAME, disrupt the CDC20-APC interaction [86,87], whereas the small molecule APC activators (M2I-1, TTKi) disrupt the CDC20-SAC interaction [88,89,90]. Protein degradation is shown by Ub, shaded with a red oval, attached to the target protein to build poly-Ub chains, followed by break down of the protein, shown in smaller circles. Inhibitory phosphorylation is shown with a red shaded “P”, and activating phosphorylation is shown with a green shaded “P”.

**Figure 2:**
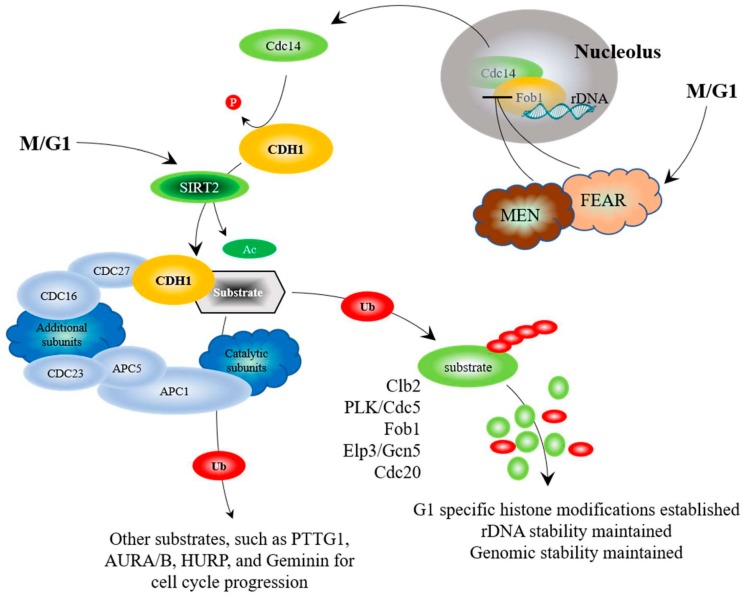
Regulation of the APC at the M/G1 transition. As mitosis comes to an end, the phosphatase Cdc14 is activated and released from sequestration within the nucleolus by Fob1, through a biphasic interaction involving the FEAR and MEN pathways [91,92]. Cdc14 dephosphorylates Cdh1, thus facilitating the interaction between Cdh1 and the APC [93]. Further activation is accomplished by deacetylation of CDH1 by SIRT2 [83]. APC^Cdh1^ function then leads to wholesale changes required for mitotic exit and transition into G1. Residual Pds1 and Clb2 are targeted for degradation by APC^Cdh1^, as are Cdc20, Cdc5, and other targets, which puts an end to the pattern of proteins required for mitotic progression [56,57,94,95,96,97,98,99,100]. Degradation of Fob1, a negative regulator of FEAR, is required for G1 progression, as Fob1 [64] is required for rDNA condensation during mitosis. Gcn5 (and likely Elp3) is also required for G1 progression [69], as it presumably acetylates histones during mitosis to establish an epigenetic pattern required for G1 progression. Once this pattern is established during mitosis, Gcn5 (and likely Elp3) must be degraded. Ubiquitinated and degraded proteins are depicted as described above.

**Figure 3:**
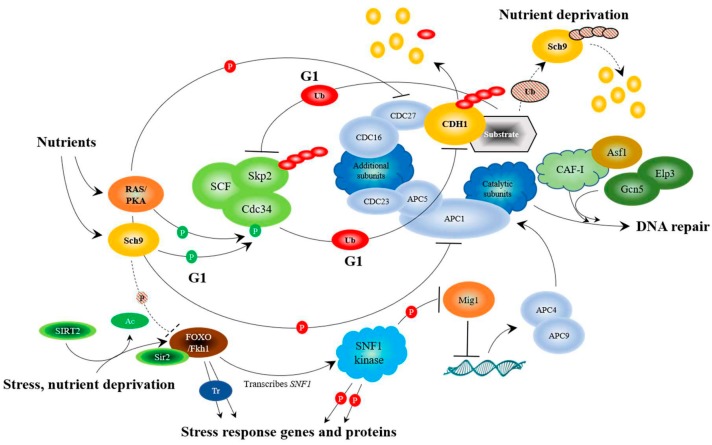
APC activity is decreased under nutrient conditions, and increased when nutrients are limiting. Inhibition of APC activity by glucose is mediated by the Ras/PKA pathway [81,101,102,103]. Recent work suggests that this is accomplished by driving the activity of the SCF ubiquitin ligase by the phosphorylation and activation of the SCF E2 component Cdc34 by the nutrient response kinases PKA and Sch9 (AKT/S6K) [104]. This could mediate APC inhibition, as it has been shown that the SCF targets the degradation of CDH1 during mitosis [58,105]. Our unpublished data also reveals that Sch9 likely inhibits Fkh1 function, and the subsequent induction of the stress pathways. Upon encountering stress, SIRT2 deacetylates and activates FOXO proteins [106], and in yeast, Sir2 physically associates with Fkh1 to facilitate inhibition of *CLB2* transcription in late M/G1 [107]. Fkh1 transcribes stress response genes (depicted by a a blue shaded “Tr”), including *SNF1*, which encodes the catalytic component of the SNF1 kinase, the yeast AMPK [79,108]. FOXO and AMPK interact across evolutionary boundaries to deal with stress [108,109,110]. The SNF1 kinase then enters the nucleus and inhibits the glucose responsive repressor Mig1, which represses the expression of the APC subunits *APC4* and *APC9* under nutrient conditions [61]. DNA repair is likely mediated, at least in part, by the APC, which controls the deposition and modification of histones during mitosis, which plays a pivotal role in DNA repair [67,69,71,72,73,74]. Inhibition of SCF-Cdc34 following APC activation is accomplished in two ways: first, the APC targets the SCF F-box protein Skp2 for degradation in G1 [111,112], and second, our unpublished data shows that the APC targets Sch9 for degradation once nutrients are depleted. Preliminary unpublished data is shown using dashed lines.

To answer these questions, it is important to identify components that connect stress and nutrient-sensing pathways. The Anaphase Promoting Complex (APC) has come to light as a potential link between the stress and nutrient sensing networks. The APC is an evolutionarily conserved large ubiquitin-protein ligase (E3) that targets proteins that inhibit mitotic entrance and exit, as well as proteins that inhibit G1 maintenance, for ubiquitin and proteasome-dependent degradation [55]. The APC is controlled by 2 co-activators, CDC20 and CDH1, which control mitotic progression, and G1 maintenance (Figure 1 and Figure 2). CDC20 binds with the APC to initiate mitosis, and is then targeted for degradation by the APC^CDH1^ complex at the M/G1 transition [56,57]. CDH1 is then targeted for degradation at the G1/S transition by a second large E3 complex called the SCF (Skp-Cullin-F-box complex) [58]. The APC is largely known for its role in cell cycle progression, but we and others have identified it as a central player in stress sensing and lifespan determination using the simple brewing yeast eukaryotic model system (Figure 3) [31,59,60,61,62,63,64,65]. Mitosis is a time during the cell cycle when DNA damage can become permanent and lead to further chromosome erosion and genomic instability [66]. The APC is also required for replication-independent chromatin assembly and histone modifications [60,67,68,69,70]. Considering that replication-independent chromatin assembly is required for DNA repair [71,72], we speculate that the APC may be involved in repair of DNA damage incurred during chromosome segregation (Figure 3). The chromatin assembly factors Asf1, and the CAF-1 complex, have been shown in yeast and human cells to be involved in assembly of histones onto repaired DNA duplexes [73,74,75,76]. The link between repair of DNA during mitosis and the APC may be the CAF-1 and Asf1 chaperones, as the APC genetically interacts with both Asf1 and CAF-1 mutants in yeast (mutant combinations have worse phenotypes), and increased expression of any one of the CAF-1 subunits, or Asf1, rescues APC defects [67]. Consistent with a role in maintaining genomic stability, APC defects result in elevated sensitivity to UV radiation, increased loss of centromere based plasmids, and increased rDNA instability [60,64].

## 8. Maintaining Genomic Stability via APC-Mediated Histone Modifications

Histone post-translational modifications are involved in cell cycle progression, particularly mitosis [113], and in DNA repair. In yeast, DNA repair requires Asf1, CAF-1, and acetylation of H3 Lys56 (H3K56^Ac^), mediated by the Asf1/Rtt109 complex [71,74]. Cells with impaired APC function have reduced H3K9^Ac^, H3K79^Me^, and H3K56^Ac^ [69]. H3K79^Me^ accumulates during mitosis [114], while H3K56^Ac^ and H3K9^Ac^ are reduced during mitosis but increase as cells enter G1 [115,116]. H3K9^Ac^ is important for transcriptional activation [117,118], H3K56^Ac^ is involved in histone deposition and DNA repair [74,119], while H3K79^Me^ is required for a variety of activities including transcriptional elongation, DNA repair, and cell cycle checkpoints [120,121]. Thus, the loss of these modifications due to impaired APC has a dramatic impact on chromatin and chromosome structure, transcription, and DNA repair. Furthermore, the histone acetyltransferase (HAT) that mediates H3K9^Ac^, Gcn5, interacts genetically and functionally with the APC [69,70]. Increased expression of *GCN5* rescued APC defects and deletion of *GCN5* in APC mutants exacerbated growth defects. Furthermore, Gcn5 is targeted by the APC for degradation at the M/G1 transition [69]. Acetylation of histones during mitosis may be important to reset the epigenome as cells re-enter G1, leading to the appropriate activation of specific genes. The correlation of Gcn5 degradation at G1, just after the accumulation of H3K9^Ac^ as cells exit mitosis, with APC mitotic function, is at the crux of establishing an active transcriptome for continued cell cycle progression. Furthermore, if targeted degradation of Gcn5 by the APC is conserved from yeast to humans, then this may be critical for tumor suppression and maintenance of genomic stability, as increased H3K9^Ac^ is associated with DNA damage, genomic instability, and progression of multiple myeloma [122]. Consistent with this, APC defects lead to elevated genomic instability in yeast [60,64,65] and in human cells [123,124]. Thus, although the APC is required for mitotic progression, it is also required to guard against damage that can occur during chromosome segregation, and to ensure that histones are acetylated to enable proper transcription as cells enter G1. These activities are all critical to ensure that cells remain healthy, leading to enhanced lifespan. On the other hand, the inability to maintain cellular homeostasis is linked with genomic instability associated with cancer development and progression

## 9. Targeting APC Inhibition for Anticancer Therapy

Because of the role the APC plays in cell cycle progression, initial work focused on the inhibition of the APC as a means to block tumor growth [125,126,127]. The evolutionarily conserved Spindle Assembly Checkpoint (SAC) complex, consisting of the proteins MAD1, MAD2, BUB1, BUBR1, BUB3 and MPS1, binds and sequesters the APC co-activator CDC20 prior to mitosis [77,78], inhibiting APC activation until all chromosomes are ready for segregation (Figure 1). It was suggested that activation of the SAC, and inhibition of the APC, would protect the cell from inappropriate chromosome segregation and mitotic catastrophe in the presence of damaged chromosomes, which is often observed in cancer cells. Furthermore, *CDC20* mRNA expression is observed to be elevated in cancer cells, which is associated with a poor prognosis; *CDC20* knockdown is required for mitotic arrest and inhibition of cell growth [94,128,129]. Specific (APCIN and pro-TAME [86,87]) and non-specific (Velcade [126]) APC inhibitors have been developed recently and inhibit tumor growth in vitro [86,130]. Both APCIN and pro-TAME act by inhibiting the interaction of CDC20 with the APC (Figure 1). Thus, inhibition of the APC was believed to be a viable anti-tumor strategy.

## 10. Targeting APC Activation for Anticancer Therapy

Recent work in mammalian cancer cells provides evidence that APC activation, rather than inhibition, may be a potent anticancer therapy that antagonizes genomic instability. As discussed above, CDC20 is an APC coactivator, and high APC^CDC20^ may be inappropriately driving cells through mitosis to promote genomic instability and cancer progression, inferring that APC inhibition will be beneficial. Regulation of CDC20 is highly coordinated (Figure 1). As discussed above, CDC20 is sequestered and inhibited by the SAC until all chromosomes are aligned along the metaphase plate and ready for segregation [77,78]. Cdc20 in yeast is activated by Cdc28-Clb2-dependent phosphorylation [80]. Cdc28-Clb2 also phosphorylates the APC subunits Cdc16, Cdc23, and Cdc27 [81], the yeast Polo-like kinase, Cdc5 [131], and Cdh1 to maintain its inactivity [82]. Once Cdc5 is activated, it then potentially targets Cdc16, Cdc27, and Apc9 for phosphorylation to further activate the APC [80,81]. Cdc5 is later targeted by APC^Cdh1^ to exit mitosis [132]. CDC20 is also deacetylated by SIRT2, adding another level of activation [83]. Additional activation signals in yeast come from the Forkhead transcription factors Fkh1 and Fkh2. The *FKH1* and *FKH2* genes are transcribed during G2 by Hcm1 [133], and are required for the transcription of the “CLB2 cluster” of genes, which contains genes required for APC activity, such as *CLB2*, *CDC5*, *CDC20,* and *APC1* [79].

**Figure 4:**
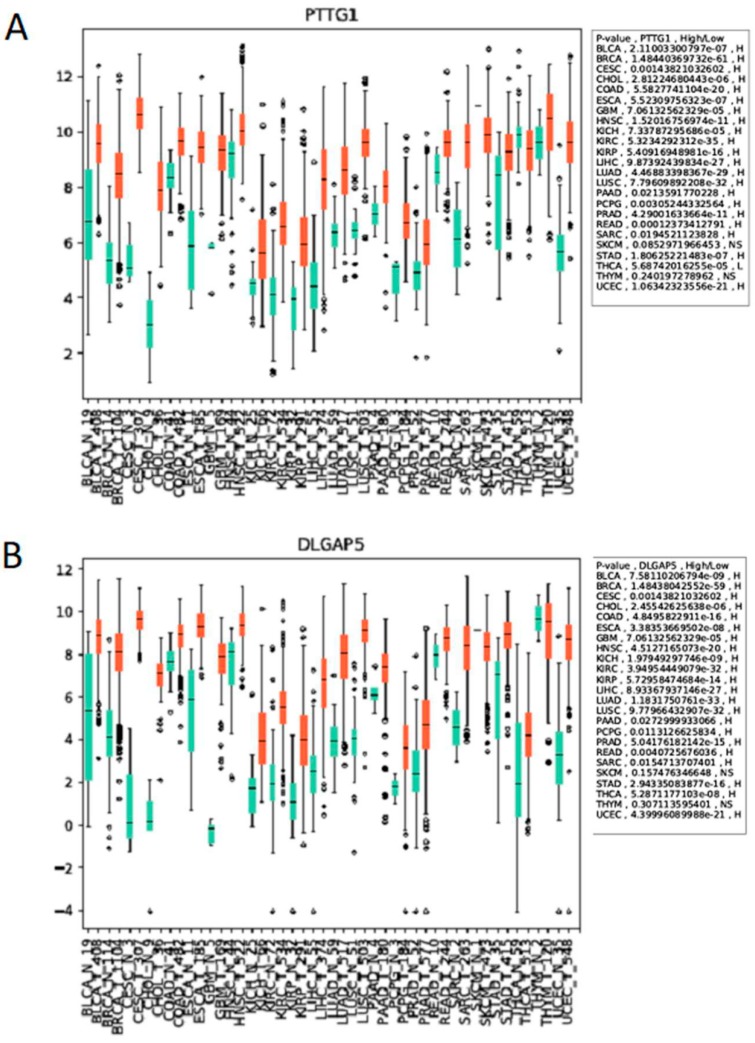
The APC substrate mRNAs PTTG1 and DLGAP5 are overexpressed in multiple cancer types. Expression scores for (**A**) PTTG1 and (**B**) DLGAP5 within 24 different types of cancer and normal tissue from TCGA [134]. The numbers in x-axis labels denote the number of patient samples in each cancer type. Statistical significance of the difference in expression between the normal and tumor samples is depicted for each cancer type. N.S. not significant. The abbreviation of each cancer in the axis label is represented as described in the TCGA portal [137].

The APC is essential, and this is conserved from yeast to humans, as yeast deletion mutants are lethal and mouse models lacking APC subunits, or CDC20, die in embryogenesis [84,134,135,136]. The APC is also essential for the prevention of aneuploidy, which contributes to tumorigenesis [84]. Thus, the systemic in vivo use of APC inhibitors may be highly toxic, limiting this approach to cancer therapy. However, an alternative interpretation is possible to explain why CDC20 accumulates in cancer cells. CDC20 itself is targeted by APC^CDH1^ for degradation once mitosis is complete [56,57]. Therefore, elevated *CDC20* expression could reflect APC^CDH1^ impairment in cancer cells, inferring that APC activation will be beneficial to cell health. Our in vitro and in vivo work (Davies, Arnason and Harkness, unpublished), and findings from others, have noted that many APC^CDH1^ mitotic substrate genes and proteins are elevated in cancer cells, including CDC20 [94], PLK1 [95], AURA/B [96,97], HURP (*DLGAP* gene [98]), Securin (*PTTG1* gene [99]), and Geminin [100], hinting that impaired APC activity as a whole is involved, rather than isolated CDC20 elevation. Moreover, using the Cancer Genome Atlas database [137], we observed that the expression of the APC substrate genes *PTTG1* and *DLGAP5* in cancer patients is differentially regulated between normal tissues and tumor tissues, across 24 different types of cancer (Figure 4).

While CDC20 has been linked to cancer progression, the second APC co-activator, CDH1, has been linked to tumor suppression, with earlier work demonstrating that cells lacking CDH1 have a shortened G1 phase, accumulate DNA damage, and undergo apoptosis [85]. CDH1 is also regulated through a complex web of interactions (Figure 2). As discussed above, the yeast Cdh1 is maintained in an inactive form by Cdc28-Clb2 phosphorylation until the end of mitosis, when Clb2 is targeted for degradation and the phosphatase Cdc14 is released from the nucleolus to undo the work of Cdc28-Clb2 [82,91,93]. The mammalian CDH1 is further activated by deacetylation by SIRT2 [83]. Recent work has demonstrated that cells with low levels of CDH1 accumulated in G1 with elevated mitotic APC substrates, causing genome instability [123,124]. Furthermore, entire loss of CDH1 increased DNA damage accumulation, driving progression of murine and human B-cell acute leukemia [138]. It was also revealed that many cancer cell lines lack the ability to activate APC^Cdh1^ when under replication stress [139,140], and that CDH1-depleted cells undergo senescence in G2, suggesting that APC^Cdh1^ may normally act as a barrier to genome instability [123]. Support for this idea comes from studies using SIRT2, an antitumor and lifespan-extending protein, which activates the APC by deacetylating CDC20 and CDH1; SIRT2-deficient mice exhibited higher levels of cancer and elevated levels of APC substrates [83]. Thus, impaired APC function appears linked with genomic instability and cancer development, providing strong therapeutic potential through targeted activation in cancer cells.

APC dysfunction and cancer development could occur in several ways. Loss of either CDC20 or CDH1 is deleterious; CDC20 deletion is lethal, while loss of CDH1 leads to genomic instability [123,124]. In addition, mutations have been observed in several APC subunit genes (APC3, APC6/CDC16, and APC8/CDC23) in cancer cells [141]. Inappropriate expression of the CDC23∆TPR mutant disrupted cell cycle progression and led to elevated levels of APC substrates. Loss of the APC7 subunit has also been implicated in various tumors [142,143]. Furthermore, silencing of a variety of APC subunits causes cells to survive treatment with compounds that inhibit the SAC, providing a mechanism for the development of drug resistance [88,89]. Thus, evidence is accumulating to support the idea that APC activity is required for cell health, while loss of normal APC function leads to genomic instability and cancer.

## 11. APC Activation Reduces Substrate Levels and Inhibits Cancer Cell Growth

Recently, focus has shifted towards the creation of compounds that activate the APC. To do so, SAC inhibition has been targeted. Prolonged SAC, or impaired APC activity, can lead to inappropriate mitotic progression in a process called mitotic slippage [144,145]. This potentially provides time for cells to respond to increased toxic levels of genomic instability common in cancer cells. Furthermore, because of the aneuploid nature of cancer cells, cancer cells are heavily reliant on the SAC for proper segregation of chromosomes; inhibition of the SAC in cancer cells produces intolerable levels of genomic instability, killing these cells [146,147]. One compound, called Mad2-inhibitor-1, or M2I-1, blocks the MAD2/CDC20 interaction (Figure 1) and weakens the SAC, leading to early activation of the APC [90]. We have subsequently used M2I-1 in vitro and in vivo, and have found that, in vitro, M2I-1 synergizes with Doxorubicin to reduce the growth of drug resistant MCF7 breast cancer cells, while growth of patient-derived triple negative breast cancer cells in mice was stalled by M2I-1 (Davies, Arnason, and Harkness, unpublished). Both in vitro and in vivo, APC substrate mRNA and protein levels were reduced, showing that M2I-1 does indeed activate the APC. Additional SAC inhibitors have been developed that inhibit the kinase MPS1/TTK (TTKi’s), a SAC component [88,89]. Kaplan-Meier plots revealed that overexpression of MPS1/TTK is correlated with poor overall and relapse-free survival in breast cancer patients [148]. Interestingly, as mentioned above, silencing of APC subunits generates resistance to the MPS1/TTK inhibitors (TTKi’s) reversine and CFI-402257 [88,89]. This suggests that the lethal mitotic segregation errors induced by TTK inhibition can be overcome by prolonging the onset of anaphase.

## 12. APC Activity, via the Fkh/SNF Kinase/Sir2 Pathway, is Required for Prolonged Longevity

We have reported that the yeast APC prolongs longevity (increased expression of only *APC10* increased replicative lifespan [61]), responds to stress, and interacts with multiple conserved stress response pathways highlighted by the Forkhead (FOXO) and Snf1 (AMPK) pathways [31,32,61,63,64,65,108] (Figure 3). It is already clear that the FOXO and AMPK pathways intersect under stress in mammalian cells and drive the activity of several other stress response networks [109,110]. In yeast, *snf1∆* mutants were also shown to interact genetically with the *apc5^CA^* mutant; deletion of *SNF1* worsened the *apc5^CA^* defect, whereas overexpression rescued it [61]. Furthermore, Mig1, a glucose responsive transcriptional repressor inhibited by Snf1 phosphorylation, repressed the expression of the APC subunits *APC4* and *APC9* [61]. Subsequent work showed that Fkh1 transcribed *SNF1*, and that increased longevity observed in the Snf1^UBA^ mutant depended on Fkh1 or Fkh2 [32]. This stress response network is further bolstered by the anti-aging protein deacetylase SIRT2, which deacetylates FOXO3a to increase its DNA binding ability in mammalian cells [106]. SIRT2 also binds to the APC^CDC20^ and APC^CDH1^ complexes and deacetylates both CDC20 and CDH1 to turn on the APC [83]. The SIRT2-FOXO interaction is also conserved in yeast, as the yeast Forkhead proteins, Fkh1 and Fkh2, physically associate with Sir2 during late M and G1 to repress the expression of the Fkh target gene Clb2 [107]. In addition, under stress conditions, Sir2 assists in APC function by inhibiting *CLB2* transcription; overexpression of *CLB2* under stress conditions is toxic [107]. However, it was not shown whether Sir2 deacetylates the Fkh proteins in this study. In yeast, the Fkh1 and Fkh2 transcription factors, like in mammalian cells, are involved in cell cycle progression, stress response, and longevity [63]. *FKH1* and *FKH2* are expressed during G2 to drive the expression of mitotic specific genes [79,149]. The *FKH* genes are activated by a third Forkhead protein called Hcm1, which is expressed at the G1/S boundary [133]. Interestingly, Hcm1 nuclear translocation is facilitated by the SNF1 kinase [150], defining a positive feedforward loop involving Snf1, Hcm1, and the Fkh proteins. Furthermore, the ubiquitin conjugating enzyme, Ubc1, interacts with the APC [151] and is required for SNF1 kinase function [108]. It was revealed that in yeast *ubc1∆* mutants, Hcm1 remains cytosolic, *FKH1* and *FKH2* transcription is reduced, and SNF1 kinase activity is decreased [108]. Fkh1 action is then reduced at the onset of mitosis, as the bulk of Fkh1 is targeted for degradation by the APC^Cdc20^ complex [65] (Figure 1). Interestingly, Fkh1 and the APC subunit Apc5 physically interacted throughout the cell cycle [65]. Deletion of both *FKH1* and *FKH2* in APC defective cells worsened the already short replicative and chronological lifespans [31], and mutation of a single, conserved lysine in Fkh1 (K_373_) mimicked the null *FKH1* allele, reduced chronological lifespan, and increased genomic instability [65]. Thus, it appears that ubiquitination of Fkh1 at K_373_, mediated by APC^Cdc20^ at the onset of mitosis, is required to maintain normal lifespan and genomic stability.

In addition to Fkh1, the APC also targets a second lifespan determinant, Fob1, for degradation [64]. Fob1 in yeast is an rDNA replication fork blocking protein [152,153]. Fob1 condenses rDNA and stalls replication fork progression during mitosis, creating free DNA ends that produce extra chromosomal circles [92,154]. Fob1 also sequesters the Cdc14 phosphatase within the nucleolus at the rDNA locus during early mitosis [91,92]. Cdc14 is released from Fob1 by the combined activity of the FEAR (Cdc14 early anaphase release) and MEN (mitotic exit network) complexes during late mitosis, enabling activation of Cdh1 via Cdc14 dephosphorylation of Cdc28-Clb2 [93]. Deletion of *FOB1* enhances yeast replicative lifespan [64,154], while increased *FOB1* expression reduces replicative lifespan [64]. We identified Fob1 as a binding partner for Apc5 in a yeast 2-hybrid screen. Mutation of an amino acid required for Fob1-Apc5 interactions (E_420_V) stabilized Fob1, increased rDNA instability, and abolished the accumulation of modified Fob1 species. We observed that Fob1 was specifically unstable during G1 and targeted for degradation by APC^Cdh1^ [64]. Deletion of *FOB1*, like that of *FKH1*, rescued the lifespan defect observed in APC mutants [64,65]. Taken together, the APC target substrates we have identified (Fkh1, Fob1, and Gcn5) function during mitosis and G1 to elicit wide-ranging effects on genomic stability and longevity (Figure 2).

## 13. The APC Triggers the End of Nutrient Signaling in the Presence of Stress

In order to fully maximize longevity, from the beginning to the end, coupling the stress and nutrient sensing pathways may be critical. The APC may be in a position to recognize both stress and nutrients. The APC is activated by phosphorylation to promote cell cycle progression. Using mouse fibroblast NIH/3T3 cells, it was shown that the Polo-like kinase, Plk, activates the APC by phosphorylating CDC16, CDC27, and APC1 [81] (Figure 1). Plk in yeast (Cdc5) also phosphorylates the APC, as does the cyclin-dependent kinase Cdc28 on Cdc16, Cdc23, and Cdc27 to activate APC^Cdc20^ function [80]. Conversely, mammalian protein kinase A (PKA) phosphorylates CDC27 and APC1 to inhibit APC function [81] (Figure 3). It is known in yeast that nutrients, such as glucose, and nutrient signaling networks involving Ras/PKA inhibit the APC [101,102,103,155]. The cell cycle proceeds in the presence of nutrients, so it remains unresolved how the positive and negative phosphorylation events on APC subunits using the nutrient response and cell cycle promoting kinases are coordinated. It remains possible that the APC’s role in cell cycle progression and stress response are controlled via different mechanisms. If this were the case, PKA inhibition of the APC may be specific to its stress response activity, whereas activation by the cyclin-dependent and Polo-like kinases may be more geared towards the APC’s cell cycle role. These observations suggest that the nutrient-sensing pathway plays a pivotal role in shutting down the APC and its stress-sensing functions.

The yeast nutrient-sensing kinases Sch9 (similar to the AKT/S6K homologues in humans [156]) and PKA also control APC activity in the presence of nutrients by phosphorylating the ubiquitin conjugating enzyme, Cdc34, the E2 component of the ubiquitin-ligase (E3) SCF [104]. Work in mammalian cells shows that the two E3 enzymes, the APC and the SCF, work to counterbalance one another during G1, with the SCF targeting the CDH1 for degradation [58,105], and the APC targeting the SCF F-box subunit SKP2 for degradation [111,112]. Thus, the nutrient response kinases inhibit APC activity in the presence of nutrients. Furthermore, our preliminary results indicate that the long life observed in *sch9∆* and *tor1∆* mutants requires functional Fkh1 or Fkh2, suggesting that Sch9 and/or Tor1 inhibit Fkh function (Postnikoff and Harkness, unpublished; Figure 3), leading to further inactivation of the APC.

However, how does the nutrient sensing pathway shut down when nutrients are limited? A recent report described the turnover of the nutrient sensing kinase Sch9 in yeast [157]. Deletion of *SCH9* in yeast increases yeast replicative and chronological lifespan [28,158], and, as mentioned above, deletion of both *FKH1* and *FKH2* in either the *sch9∆* or *tor1∆* background eliminates the observed long life (Postnikoff and Harkness, unpublished). As cells entered stationary phase, it was observed that total ubiquitinated protein decreased, as did total Sch9 protein levels [157]. In the presence of the proteasome poison MG132, it was observed that Sch9 protein levels increased [157], supporting the idea that Sch9 is ubiquitinated and degraded as nutrient levels decrease. We therefore asked whether Sch9 is targeted for ubiquitination by the APC, as a means to inactivate this arm of the nutrient response network when nutrient levels decline. Our preliminary experiments show that deletion of *SCH9* in APC mutants suppressed the chronological lifespan and oxidative stress sensitive defects in APC mutants (Postnikoff and Harkness, unpublished). We also confirmed that Sch9 turnover occurs as cells enter stationary phase, and that this is blocked in APC mutants (Malo and Harkness, unpublished). Taken as a whole, the published and unpublished literature supports the idea that the APC sits at the apex of the stress and nutrient-sensing pathways, controlling cell cycle progression, DNA repair, and chromosome maintenance (Figure 3).

## 14. Conclusions

The positioning of the APC at the intersection point of the stress and nutrient sensing pathways confers importance upon this complex, as it may have the potential to protect the cells that come together to form the zygote from the aging process. The potential for aging likely begins for an individual as soon as the germ cells responsible for them are born. For the oocyte, that means during the mother’s in utero development. It will be many years before that oocyte is fertilized; therefore, plenty of time exists for damaging side effects of cell metabolism to rear their ugly heads. It is critical that the repair mechanisms within these cells are functioning optimally. As long as the APC is at its peak function, protection against cellular damage should be high. With continued proper function of the APC through the life of the germ cells and the subsequent offspring, increased healthspan may be possible.

## References

[1] Sobel H. (1966). When does human aging start?. Gerontologist.

[2] Lakatta E.G. (2015). So! What’s aging? Is cardiovascular aging a disease?. J. Mol. Cell. Cardiol..

[3] Paashuis-Lew Y.R., Heddle J.A. (1998). Spontaneous mutation during fetal development and post-natal growth. Mutagenesis.

[4] Relton C.L., Daniel C.P., Hammal D.M., Parker L., Janet Tawn E., Burn J. (2004). DNA repair gene polymorphisms, pre-natal factors and the frequency of somatic mutations in the glycophorin-A gene among healthy newborns. Mutat. Res..

[5] Milne E.M. (2006). When does human ageing begin?. Mech. Ageing Dev..

[6] Kubben N., Misteli T. (2017). Shared molecular and cellular mechanisms of premature ageing and ageing-associated diseases. Nat. Rev. Mol. Cell Biol..

[7] Hayflick L. (1984). When does aging begin?. Res. Aging.

[8] Hayflick L. (2000). The future of ageing. Nature.

[9] Hayflick L. (2004). The not-so-close relationship between biological aging and age-associated pathologies in humans. J. Gerontol. A Biol. Sci. Med. Sci..

[10] Kirkwood T.B., Austad S.N. (2000). Why do we age?. Nature.

[11] Nemoto S., Finkel T. (2004). Ageing and the mystery at Arles. Nature.

[12] Harman D. (1956). Aging: A theory based on free radical and radiation chemistry. J. Gerontol..

[13] Hayflick L. (2007). Entropy explains aging, genetic determination explains longevity, and undefined terminology explains misunderstanding both. PLoS Genet..

[14] Labbadia J., Morimoto R.I. (2014). Proteostasis and longevity: When does aging really begin?. F1000Prime Rep..

[15] Kirkwood T.B., Holliday R. (1979). The evolution of ageing and longevity. Proc. R. Soc. Lond. B Biol. Sci..

[16] Kenyon C.J. (2010). The genetics of ageing. Nature.

[17] Salminen A., Kaarniranta K. (2010). ER stress and hormetic regulation of the aging process. Ageing Res. Rev..

[18] Martins I., Galluzzi L., Kroemer G. (2011). Hormesis, cell death and aging. Aging.

[19] Williams G.C. (1957). Pleiotrophy, natural selection and the evolution of senescence. Evolution.

[20] Kirkwood T.B., Rose M.R. (1991). Evolution of senescence: Late survival sacrificed for reproduction. Philos. Trans. R Soc. Lond. B Biol. Sci..

[21] Nesse R.M., Williams G.C. (1998). Evolution and the origins of disease. Sci. Am..

[22] Wick G., Berger P., Jansen-Dürr P., Grubeck-Loebenstein B.A. (2003). Darwinian-evolutionary concept of age-related diseases. Exp. Gerontol..

[23] Artandi S.E., DePinho R.A. (2000). A critical role for telomeres in suppressing and facilitating carcinogenesis. Curr. Opin. Genet. Dev..

[24] Shay J.W., Wright W.E. (2001). Ageing and cancer: The telomere and telomerase connection. Novartis Found. Symp..

[25] López-Otín C., Blasco M.A., Partridge L., Serrano M., Kroemer G. (2013). The hallmarks of aging. Cell.

[26] Aunan J.R., Watson M.M., Hagland H.R., Søreide K. (2016). Molecular and biological hallmarks of ageing. Br. J. Surg..

[27] Kaeberlein M., McVey M., Guarente L. (1999). The SIR2/3/4 complex and SIR2 alone promote longevity in *Saccharomyces cerevisiae* by two different mechanisms. Genes Dev..

[28] Fabrizio P., Pozza F., Pletcher S.D., Gendron C.M., Longo V.D. (2001). Regulation of longevity and stress resistance by Sch9 in yeast. Science.

[29] Wei M., Fabrizio P., Hu J., Ge H., Cheng C., Li L., Longo V.D. (2008). Life span extension by calorie restriction depends on Rim15 and transcription factors downstream of Ras/PKA, Tor, and Sch9. PLoS Genet..

[30] Lu J.Y., Lin Y.Y., Sheu J.C., Wu J.T., Lee F.J., Chen Y., Lin M.I., Chiang F.T., Tai T.Y., Berger S.L. (2011). Acetylation of yeast AMPK controls intrinsic aging independently of caloric restriction. Cell.

[31] Postnikoff S.D., Malo M.M., Wong B., Harkness T.A. (2012). The yeast forkhead transcription factors fkh1 and fkh2 regulate lifespan and stress response together with the anaphase-promoting complex. PLoS Genet..

[32] Jiao R., Postnikoff S., Harkness T.A., Arnason T.G. (2015). The SNF1 Kinase Ubiquitin-associated Domain Restrains Its Activation, Activity, and the Yeast Life Span. J. Biol. Chem..

[33] Mirisola M.G., Taormina G., Fabrizio P., Wei M., Hu J., Longo V.D. (2014). Serine- and threonine/valine-dependent activation of PDK and Tor orthologs converge on Sch9 to promote aging. PLoS Genet..

[34] Dong X., Milholland B., Vijg J. (2016). Evidence for a limit to human lifespan. Nature.

[35] Robine J.M., Allard M. (1998). The oldest human. Science.

[36] Weon B.M., Je J.H. (2009). Theoretical estimation of maximum human lifespan. Biogerontology.

[37] Modig K., Andersson T., Vaupel J., Rau R., Ahlbom A. (2017). How long do centenarians survive? Life expectancy and maximum lifespan. J. Intern. Med..

[38] Gerontology Research Group. http://supercentenarian-research-foundation.org/TableE.aspx.

[39] De Grey A. (2005). A strategy for postponing aging indefinitely. Stud. Health Technol. Inform..

[40] Lenart A., Vaupel J.W. (2017). Questionable evidence for a limit to human lifespan. Nature.

[41] Hughes B.G., Hekimi S. (2017). Many possible maximum lifespan trajectories. Nature.

[42] Takahashi Y., Kuro-o M., Ishikawa F. (2000). Aging mechanisms. Proc. Natl. Acad. Sci. USA.

[43] Lucke J.C., Hall W. (2005). Who wants to live forever?. EMBO Rep..

[44] Tacutu R., Thornton D., Johnson E., Budovsky A., Barardo D., Craig T., Diana E., Lehmann G., Toren D., Wang J. (2018). Human Ageing Genomic Resources: New and updated databases. Nucleic Acids Res..

[45] Lindahl T. (1993). Instability and decay of the primary structure of DNA. Nature.

[46] Hoeijmakers J.H. (2009). DNA damage, aging, and cancer. N. Engl. J. Med..

[47] Cervelli T., Borghini A., Galli A., Andreassi M.G. (2012). DNA damage and repair in atherosclerosis: Current insights and future perspectives. Int. J. Mol. Sci..

[48] Vijg J., Suh Y. (2013). Genome instability and aging. Annu. Rev. Physiol..

[49] Grindel A., Brath H., Nersesyan A., Knasmueller S., Wagner K.H. (2017). Association of genomic instability with HbA1c levels and medication in diabetic patients. Sci. Rep..

[50] Yuza K., Nagahashi M., Watanabe S., Takabe K., Wakai T. (2017). Hypermutation and microsatellite instability in gastrointestinal cancers. Oncotarget.

[51] Barzilai A., Schumacher B., Shiloh Y. (2017). Genome instability: Linking ageing and brain degeneration. Mech. Ageing Dev..

[52] Henderson K.A., Gottschling D.E. (2008). A mother’s sacrifice: what is she keeping for herself?. Curr. Opin. Cell Biol..

[53] Gottschling D.E., Nyström T. (2017). The Upsides and Downsides of Organelle Interconnectivity. Cell.

[54] Sun Y.C., Sun X.F., Dyce P.W., Shen W., Chen H. (2017). The role of germ cell loss during primordial follicle assembly: A review of current advances. Int. J. Biol. Sci..

[55] Sivakumar S., Gorbsky G.J. (2015). Spatiotemporal regulation of the anaphase-promoting complex in mitosis. Nat. Rev. Mol. Cell Biol..

[56] Zhang S., Chang L., Alfieri C., Zhang Z., Yang J., Maslen S., Skehel M., Barford D. (2016). Molecular mechanism of APC/C activation by mitotic phosphorylation. Nature.

[57] Zhou Z., He M., Shah A.A., Wan Y. (2016). Insights into APC/C: From cellular function to diseases and therapeutics. Cell Div..

[58] Fukushima H., Ogura K., Wan L., Lu Y., Li V., Gao D., Liu P., Lau A.W., Wu T., Kirschner M.W. (2013). SCF-mediated Cdh1 degradation defines a negative feedback system that coordinates cell-cycle progression. Cell Rep..

[59] Simpson-Lavy K.J., Sajman J., Zenvirth D., Brandeis M. (2009). APC/CCdh1 specific degradation of Hsl1 and Clb2 is required for proper stress responses of *S. cerevisiae*. Cell Cycle.

[60] Harkness T.A., Davies G.F., Ramaswamy V., Arnason T.G. (2002). The ubiquitin-dependent targeting pathway in Saccharomyces cerevisiae plays a critical role in multiple chromatin assembly regulatory steps. Genetics.

[61] Harkness T.A., Shea K.A., Legrand C., Brahmania M., Davies G.F. (2004). A functional analysis reveals dependence on the Anaphase Promoting Complex for prolonged life span in yeast. Genetics.

[62] Lindsay D.L., Bonham-Smith P.C., Postnikoff S., Gray G.R., Harkness T.A. (2011). A role for the anaphase promoting complex in hormone regulation. Planta.

[63] Postnikoff S.D., Harkness T.A. (2012). Mechanistic insights into aging, cell-cycle progression, and stress response. Front. Physiol..

[64] Menzel J., Malo M.E., Chan C., Prusinkiewicz M., Arnason T.G., Harkness T.A. (2014). The anaphase promoting complex regulates yeast lifespan and rDNA stability by targeting Fob1 for degradation. Genetics.

[65] Malo M.E., Postnikoff S.D., Arnason T.G., Harkness T.A. (2016). Mitotic degradation of yeast Fkh1 by the Anaphase Promoting Complex is required for normal longevity, genomic stability and stress resistance. Aging.

[66] Ganem N.J., Pellman D. (2012). Linking abnormal mitosis to the acquisition of DNA damage. J. Cell Biol..

[67] Harkness T.A., Arnason T.G., Legrand C., Pisclevich M.G., Davies G.F., Turner E.L. (2005). Contribution of CAF-I to anaphase-promoting-complex-mediated mitotic chromatin assembly in *Saccharomyces cerevisiae*. Eukaryot Cell.

[68] Arnason T.G., Pisclevich M.G., Dash M.D., Davies G.F., Harkness T.A. (2005). Novel interaction between Apc5p and Rsp5p in an intracellular signaling pathway in *Saccharomyces cerevisiae*. Eukaryot Cell.

[69] Turner E.L., Malo M.E., Pisclevich M.G., Dash M.D., Davies G.F., Arnason T.G., Harkness T.A. (2010). The *Saccharomyces cerevisiae* Anaphase Promoting Complex interacts with multiple histone-modifying enzymes to regulate cell cycle progression. Eukaryot Cell.

[70] Islam A., Turner E.L., Menzel J., Malo M.E., Harkness T.A. (2011). Antagonistic Gcn5-Hda1 interactions revealed by mutations to the Anaphase Promoting Complex in yeast. Cell Div..

[71] Linger J.G., Tyler J.K. (2007). Chromatin Disassembly and Reassembly during DNA Repair. Mutat. Res..

[72] Li X., Tyler J.K. (2016). Nucleosome disassembly during human non-homologous end joining followed by concerted HIRA- and CAF-1-dependent reassembly. eLife.

[73] Mello J.A., Silljé H.H.W., Roche D.M.J., Kirschner D.B., Nigg E.A., Almouzni G. (2002). Human Asf1 and CAF-1 interact and synergize in a repair-coupled nucleosome assembly pathway. EMBO Rep..

[74] Chen C.C., Tyler J. (2008). Chromatin reassembly signals the end of DNA repair. Cell Cycle.

[75] Kim J.A., Haber J.E. (2009). Chromatin assembly factors Asf1 and CAF-1 have overlapping roles in deactivating the DNA damage checkpoint when DNA repair is complete. Proc. Natl. Acad. Sci. USA.

[76] Rodriges Blanko E., Kadyrova L.Y., Kadyrov F.A. (2016). DNA Mismatch Repair Interacts with CAF-1- and ASF1A-H3-H4-dependent Histone (H3-H4)_2_ Tetramer Deposition. J. Biol. Chem..

[77] Izawa D., Pines J. (2015). The mitotic checkpoint complex binds a second CDC20 to inhibit active APC/C. Nature.

[78] Alfieri C., Chang L., Zhang Z., Yang J., Maslen S., Skehel M., Barford D. (2016). Molecular basis of APC/C regulation by the spindle assembly checkpoint. Nature.

[79] Zhu G., Spellman P.T., Volpe T., Brown P.O., Botstein D., Davis T.N., Futcher B. (2000). Two yeast forkhead genes regulate the cell cycle and pseudohyphal growth. Nature.

[80] Rudner A.D., Murray A.W. (2000). Phosphorylation by Cdc28 activates the Cdc20-dependent activity of the anaphase-promoting complex. J. Cell Biol..

[81] Kotani S., Tugendreich S., Fujii M., Jorgensen P.M., Watanabe N., Hoog C., Hieter P., Todokoro K. (1998). PKA and MPF-activated polo-like kinase regulate anaphase-promoting complex activity and mitosis progression. Mol. Cell.

[82] Crasta K., Lim H.H., Giddings T.H., Winey M., Surana U. (2008). Inactivation of Cdh1 by synergistic action of Cdk1 and polo kinase is necessary for proper assembly of the mitotic spindle. Nat. Cell Biol..

[83] Kim H.S., Vassilopoulos A., Wang R.H., Lahusen T., Xiao Z. (2011). SIRT2 maintains genome integrity and suppresses tumorigenesis through regulating APC/C activity. Cancer Cell.

[84] Zhang J., Wan L., Dai X., Sun Y., Wei W. (2014). Functional characterization of Anaphase Promoting Complex/Cyclosome (APC/C) E3 ubiquitin ligases in tumorigenesis. Biochim. Biophys. Acta.

[85] Peters J.M. (2006). The anaphase promoting complex/cyclosome: A machine designed to destroy. Nat. Rev. Mol. Cell Biol..

[86] Sackton K.L., Dimova N., Zeng X., Tian W., Zhang M., Sackton T.B., Meaders J., Pfaff K.L., Sigoillot F., Yu H. (2014). Synergistic blockade of mitotic exit by two chemical inhibitors of the APC/C. Nature.

[87] Wang L., Zhang J., Wan L., Zhou X., Wang Z., Wei W. (2015). Targeting Cdc20 as a novel cancer therapeutic strategy. Pharmacol. Ther..

[88] Sansregret L., Patterson J.O., Dewhurst S., López-García C., Koch A., McGranahan N., Chao W.C.H., Barry D.J., Rowan A., Instrell R. (2017). APC/C Dysfunction Limits Excessive Cancer Chromosomal Instability. Cancer Discov..

[89] Thu K.L., Silvester J., Elliott M.J., Ba-Alawi W., Duncan M.H., Elia A.C., Mer A.S., Smirnov P., Safikhani Z., Haibe-Kains B. (2018). Disruption of the anaphase-promoting complex confers resistance to TTK inhibitors in triple-negative breast cancer. Proc. Natl. Acad. Sci. USA.

[90] Kastl J., Braun J., Prestel A., Möller H.M., Huhn T., Mayer T.U. (2015). Mad2 Inhibitor-1 (M2I-1): A Small Molecule Protein-Protein Interaction Inhibitor Targeting the Mitotic Spindle Assembly Checkpoint. ACS Chem. Biol..

[91] Stegmeier F., Huang J., Rahal R., Zmolik J., Moazed D., Amon A. (2004). The replication fork block protein Fob1 functions as a negative regulator of the FEAR network. Curr. Biol..

[92] Waples W.G., Chahwan C., Ciechonska M., Lavoie B.D. (2009). Putting the brake on FEAR: Tof2 promotes the biphasic release of Cdc14 phosphatase during mitotic exit. Mol. Biol. Cell.

[93] Visintin R., Craig K., Hwang E.S., Prinz S., Tyers M., Amon A. (1998). The phosphatase Cdc14 triggers mitotic exit by reversal of Cdk-dependent phosphorylation. Mol. Cell.

[94] Karra H., Repo H., Ahonen I., Löyttyniemi E., Pitkänen R., Lintunen M., Kuopio T., Söderström M., Kronqvist P. (2014). Cdc20 and securin overexpression predict short-term breast cancer survival. Br. J. Cancer.

[95] Schmit T.L., Ledesma M.C., Ahmad N. (2010). Modulating polo-like kinase 1 as a means for cancer chemoprevention. Pharm. Res..

[96] Staff S., Isola J., Jumppanen M., Tanner M. (2010). Aurora-A gene is frequently amplified in basal-like breast cancer. Oncol. Rep..

[97] Heredia F.F., de Sousa J.C., Ribeiro Junior H.L., Carvalho A.F., Magalhaes S.M., Pinheiro R.F. (2014). Proteins related to the spindle and checkpoint mitotic emphasize the different pathogenesis of hypoplastic MDS. Leuk. Res..

[98] Kuo T.C., Lu H.P., Chao C.C. (2011). The tyrosine kinase inhibitor sorafenib sensitizes hepatocellular carcinoma cells to taxol by suppressing the HURP protein. Biochem. Pharmacol..

[99] Xiang W., Wu X., Huang C., Wang M., Zhao X., Luo G., Li Y., Jiang G., Xiao X., Zeng F. (2017). PTTG1 regulated by miR-146a-3p promotes bladder cancer migration, invasion, metastasis and growth. Oncotarget.

[100] Zhang L., Cai M., Gong Z., Zhang B., Li Y., Guan L., Hou X., Li Q., Liu G., Xue Z. (2017). Geminin facilitates FoxO3 deacetylation to promote breast cancer cell metastasis. J. Clin. Investig..

[101] Irniger S., Bäumer M., Braus G.H. (2000). Glucose and ras activity influence the ubiquitin ligases APC/C and SCF in *Saccharomyces cerevisiae*. Genetics.

[102] Bolte M., Dieckhoff P., Krause C., Braus G.H., Irniger S. (2003). Synergistic inhibition of APC/C by glucose and activated Ras proteins can be mediated by each of the Tpk1-3 proteins in *Saccharomyces cerevisiae*. Microbiology.

[103] Searle J.S., Schollaert K.L., Wilkins B.J., Sanchez Y. (2004). The DNA damage checkpoint and PKA pathways converge on APC substrates and Cdc20 to regulate mitotic progression. Nat. Cell Biol..

[104] Cocklin R., Goebl M. (2011). Nutrient sensing kinases PKA and Sch9 phosphorylate the catalytic domain of the ubiquitin-conjugating enzyme Cdc34. PLoS ONE.

[105] Choudhury R., Bonacci T., Arceci A., Lahiri D., Mills C.A., Kernan J.L., Branigan T.B., DeCaprio J.A., Burke D.J., Emanuele M.J. (2016). APC/C and SCF(cyclin F) constitute a reciprocal feedback circuit controlling S-phase entry. Cell Rep..

[106] Wang F., Nguyen M., Qin F.X., Tong Q. (2007). SIRT2 deacetylates FOXO3a in response to oxidative stress and caloric restriction. Aging Cell.

[107] Linke C., Klipp E., Lehrach H., Barberis M., Krobitsch S. (2013). Fkh1 and Fkh2 associate with Sir2 to control CLB2 transcription under normal and oxidative stress conditions. Front. Physiol..

[108] Jiao R., Lobanova L., Waldner A., Fu A., Xiao L., Harkness T.A., Arnason T.G. (2016). The ubiquitin-conjugating enzyme, Ubc1, indirectly regulates SNF1 kinase activity via Forkhead-dependent transcription. Microb. Cell.

[109] Chiacchiera F., Simone C. (2010). The AMPK-FoxO3A axis as a target for cancer treatment. Cell Cycle.

[110] Salminen A., Kaarniranta K. (2012). AMP-activated protein kinase (AMPK) controls the aging process via an integrated signaling network. Ageing Res. Rev..

[111] Wei W., Ayad N.G., Wan Y., Zhang G.J., Kirschner M.W., Kaelin W.G. (2004). Degradation of the SCF component Skp2 in cell-cycle phase G1 by the anaphase-promoting complex. Nature.

[112] Bashir T., Dorrello N.V., Amador V., Guardavaccaro D., Pagano M. (2004). Control of the SCF(Skp2-Cks1) ubiquitin ligase by the APC/C(Cdh1) ubiquitin ligase. Nature.

[113] Wang F., Higgins J.M. (2013). Histone modifications and mitosis: countermarks, landmarks, and bookmarks. Trends Cell Biol..

[114] Guppy B.J., McManus K.J. (2015). Mitotic accumulation of dimethylated lysine 79 of histone H3 is important for maintaining genome integrity during mitosis in human cells. Genetics.

[115] Recht J., Tsubota T., Tanny J.C., Diaz R.L., Berger J.M., Zhang X., Garcia B.A., Shabanowitz J., Burlingame A.L., Hunt D.F. (2006). Histone chaperone Asf1 is required for histone H3 lysine 56 acetylation, a modification associated with S phase in mitosis and meiosis. Proc. Natl. Acad. Sci. USA.

[116] Jeong Y.S., Cho S., Park J.S., Ko Y., Kang Y.K. (2010). Phosphorylation of serine-10 of histone H3 shields modified lysine-9 selectively during mitosis. Genes Cells.

[117] Karmodiya K., Krebs A.R., Oulad-Abdelghani M., Kimura H., Tora L.H. (2012). 3K9 and H3K14 acetylation co-occur at many gene regulatory elements, while H3K14ac marks a subset of inactive inducible promoters in mouse embryonic stem cells. BMC Genom..

[118] Gates L.A., Shi J., Rohira A.D., Feng Q., Zhu B., Bedford M.T., Sagum C.A., Jung S.Y., Qin J., Tsai M.J. (2017). Acetylation on histone H3 lysine 9 mediates a switch from transcription initiation to elongation. J. Biol. Chem..

[119] Downs J.A. (2008). Histone H3 K56 acetylation, chromatin assembly, and the DNA damage checkpoint. DNA Repair.

[120] Farooq Z., Banday S., Pandita T.K., Altaf M. (2016). The many faces of histone H3K79 methylation. Mutat. Res. Rev. Mutat. Res..

[121] Wood K., Tellier M., Murphy S. (2018). DOT1L and H3K79 Methylation in Transcription and Genomic Stability. Biomolecules.

[122] Cea M., Cagnetta A., Adamia S., Acharya C., Tai Y.T., Fulciniti M., Ohguchi H., Munshi A., Acharya P., Bhasin M.K. (2016). Evidence for a role of the histone deacetylase SIRT6 in DNA damage response of multiple myeloma cells. Blood.

[123] Ercilla A., Llopis A., Feu S., Aranda S., Ernfors P. (2016). New origin firing is inhibited by APC/CCdh1 activation in S-phase after severe replication stress. Nucleic Acids Res..

[124] Garzón J., Rodríguez R., Kong Z., Chabes A., Rodríguez-Acebes S., Méndez J., Moreno S., García-Higuera I. (2017). Shortage of dNTPs underlies altered replication dynamics and DNA breakage in the absence of the APC/C cofactor Cdh1. Oncogene.

[125] Wäsch R., Engelbert D. (2005). Anaphase-promoting complex-dependent proteolysis of cell cycle regulators and genomic instability of cancer cells. Oncogene.

[126] Cardozo T., Pagano M. (2007). Wrenches in the works: Drug discovery targeting the SCF ubiquitin ligase and APC/C complexes. BMC Biochem..

[127] Bolanos-Garcia V.M. (2009). Assessment of the mitotic spindle assembly checkpoint (SAC) as the target of anticancer therapies. Curr. Cancer Drug Targets.

[128] Taniguchi K., Momiyama N., Ueda M., Matsuyama R., Mori R., Fujii Y., Ichikawa Y., Endo I., Togo S., Shimada H. (2008). Targeting of CDC20 via Small Interfering RNA Causes Enhancement of the Cytotoxicity of Chemoradiation. Anticancer Res..

[129] Chang D.Z., Ma Y., Ji B., Liu Y., Hwu P., Abbruzzese J.L., Logsdon C., Wang H. (2012). Increased CDC20 expression is associated with pancreatic ductal adenocarcinoma differentiation and progression. J. Hematol. Oncol..

[130] Lub S., Maes A., Maes K., De Veirman K., De Bruyne E., Menu E., Fostier K., Kassambara A., Moreaux J., Hose D. (2016). Inhibiting the anaphase promoting complex/cyclosome induces a metaphase arrest and cell death in multiple myeloma cells. Oncotarget.

[131] Mortensen E.M., Haas W., Gygi M., Gygi S.P., Kellogg D.R. (2005). Cdc28-dependent regulation of the Cdc5/Polo kinase. Curr. Biol..

[132] Visintin C., Tomson B.N., Rahal R., Paulson J., Cohen M., Taunton J., Amon A., Visintin R. (2008). APC/C-Cdh1-mediated degradation of the Polo kinase Cdc5 promotes the return of Cdc14 into the nucleolus. Genes Dev..

[133] Pramila T., Wu W., Miles S., Noble W.S., Breeden L.L. (2006). The Forkhead transcription factor Hcm1 regulates chromosome segregation genes and fills the S-phase gap in the transcriptional circuitry of the cell cycle. Genes Dev..

[134] Lamb J.R., Michaud W.A., Sikorski R.S., Hieter P.A. (1994). Cdc16p, Cdc23p and Cdc27p form a complex essential for mitosis. EMBO J..

[135] Irniger S., Nasmyth K. (1997). The anaphase-promoting complex is required in G1 arrested yeast cells to inhibit B-type cyclin accumulation and to prevent uncontrolled entry into S-phase. J. Cell Sci..

[136] Li M., York J.P., Zhang P. (2007). Loss of Cdc20 causes a securin-dependent metaphase arrest in two-cell mouse embryos. Mol. Cell. Biol..

[137] TCGA. https://portal.gdc.cancer.gov.

[138] Ishizawa J., Sugihara E., Kuninaka S., Mogushi K., Kojima K., Benton C.B., Zhao R., Chachad D., Hashimoto N., Jacamo R.O. (2017). FZR1 loss increases sensitivity to DNA damage and consequently promotes murine and human B-cell acute leukemia. Blood.

[139] Villa-Hernández S., Bueno A., Bermejo R. (2017). The Multiple Roles of Ubiquitylation in Regulating Challenged DNA Replication. Adv. Exp. Med. Biol..

[140] Kitao H., Iimori M., Kataoka Y., Wakasa T., Tokunaga E. (2018). DNA replication stress and cancer chemotherapy. Cancer Sci..

[141] Wang Q., Moyret-Lalle C., Couzon F., Surbiguet-Clippe C., Saurin J.C., Lorca T., Navarro C., Puisieux A. (2003). Alterations of anaphase-promoting complex genes in human colon cancer cells. Oncogene.

[142] Park K.H., Choi S.E., Eom M., Kang Y. (2005). Downregulation of the anaphase-promoting complex (APC)7 in invasive ductal carcinomas of the breast and its clinicopathologic relationships. Breast Cancer Res..

[143] Kim I.Y., Kwon H.Y., Park K.H., Kim D.S. (2017). Anaphase-Promoting Complex 7 is a Prognostic Factor in Human Colorectal Cancer. Ann. Coloproctol..

[144] Tsuda Y., Iimori M., Nakashima Y., Nakanishi R., Ando K., Ohgaki K., Kitao H., Saeki H., Oki E., Maehara Y. (2017). Mitotic slippage and the subsequent cell fates after inhibition of Aurora B during tubulin-binding agent-induced mitotic arrest. Sci. Rep..

[145] Haschka M., Karbon G., Fava L.L., Villunger A. (2018). Perturbing mitosis for anti-cancer therapy: Is cell death the only answer?. EMBO Rep..

[146] Kops G.J., Foltz D.R., Cleveland D.W. (2004). Lethality to human cancer cells through massive chromosome loss by inhibition of the mitotic checkpoint. Proc. Natl. Acad. Sci. USA.

[147] Bharadwaj R., Yu H. (2004). The spindle checkpoint, aneuploidy, and cancer. Oncogene.

[148] Lee M., Rivera-Rivera Y., Moreno C.S., Saavedra H.I. (2017). The E2F activators control multiple mitotic regulators and maintain genomic integrity through Sgo1 and BubR1. Oncotarget.

[149] Hollenhorst P.C., Bose M.E., Mielke M.R., Müller U., Fox C.A. (2000). Forkhead genes in transcriptional silencing, cell morphology and the cell cycle. Overlapping and distinct functions for FKH1 and FKH2 in *Saccharomyces cerevisiae*. Genetics.

[150] Rodríguez-Colman M.J., Sorolla M.A., Vall-Llaura N., Tamarit J., Ros J., Cabiscol E. (2013). The FOX transcription factor Hcm1 regulates oxidative metabolism in response to early nutrient limitation in yeast. Role of Snf1 and Tor1/Sch9 kinases. Biochim. Biophys. Acta.

[151] Rodrigo-Brenni M.C., Morgan D.O. (2007). Sequential E2s drive polyubiquitin chain assembly on APC targets. Cell.

[152] Kobayashi T., Horiuchi T. (1996). A yeast gene product, Fob1 protein, required for both replication fork blocking and recombinational hotspot activities. Genes Cells.

[153] Kobayashi T., Heck D.J., Nomura M., Horiuchi T. (1998). Expansion and contraction of ribosomal DNA repeats in *Saccharomyces cerevisiae*: Requirement of replication fork blocking (Fob1) protein and the role of RNA polymerase I. Genes Dev..

[154] Defossez P.A., Prusty R., Kaeberlein M., Lin S.J., Ferrigno P., Silver P.A., Keil R.L., Guarente L. (1999). Elimination of replication block protein Fob1 extends the life span of yeast mother cells. Mol. Cell.

[155] Yamashita Y.M., Nakaseko Y., Samejima I., Kumada K., Yamada H., Michaelson D., Yanagida M. (1996). 20S cyclosome complex formation and proteolytic activity inhibited by the cAMP/PKA pathway. Nature.

[156] Madia F., Gattazzo C., Wei M., Fabrizio P., Burhans W.C., Weinberger M., Galbani A., Smith J.R., Nguyen C., Huey S. (2008). Longevity mutation in SCH9 prevents recombination errors and premature genomic instability in a Werner/Bloom model system. J. Cell Biol..

[157] Qie B., Lyu Z., Lyu L., Liu J., Gao X., Liu Y., Duan W., Zhang N., Du L., Liu K. (2015). Sch9 regulates intracellular protein ubiquitination by controlling stress responses. Redox Biol..

[158] Kaeberlein M., Powers R.W., Steffen K.K., Westman E.A., Hu D., Dang N., Kerr E.O., Kirkland K.T., Fields S., Kennedy B.K. (2005). Regulation of yeast replicative life span by TOR and Sch9 in response to nutrients. Science.

